# Carcinome neuroendocrine du sein: à propos d'un cas et revue de la littérature

**Published:** 2012-10-29

**Authors:** Hinde El Fatemi, Nawal Hammas, Kaoutar Moumna, Mouhcine Bedahou, Nawfel Mellas, Omar Mesbahi

**Affiliations:** 1 Laboratoire d'anatomie et cytologie pathologique, CHU HASSAN II, Fès, Maroc; 2 Service d'oncologie médicale, CHU HASSAN II, Fès, Maroc

**Keywords:** Carcinome neuro-endocrine, cancer du sein, chromogranine, immunohistochimie, neuroendocrine carcinoma, breast cancer, chromogranin, immunohistochemistry

## Abstract

Le carcinome neuroendocrine primitif du sein est une tumeur rare qui a été reconnue par la dernière édition de la classification OMS du cancer du sein publiée en 2003. Le diagnostic est évoqué sur des critères morphologiques et confirmé par l'expression des marqueurs neuroendocrines (chromogranine et synaptophysine) par plus de 50% des cellules tumorales. Nous rapportons un nouveau cas de carcinome neuroendocrine primitif du sein, et à travers une revue de la littérature, nous détaillons les aspects épidémiologiques, morphologiques et immuno-histochimiques de cette tumeur rare.

## Introduction

Les carcinomes neuroendocrines touchent essentiellement le système bronchopulmonaire et le tractus gastro-intestinal. Les localisations mammaires sont rares [1, 2]. Elles représentent moins de 0.1% de tous les cancers mammaires et moins de 1% des tumeurs neuro-endocrines. [1]. Le carcinome neuro-endocrine mammaire était initialement décrit par Cubilla et al. en 1977; depuis d'autres cas ont été rapportés [2]. Nous rapportons un nouveau cas de carcinome neuroendocrine primitif du sein, et à travers une revue de la littérature, nous détaillons les aspects épidémiologiques, morphologiques et immuno-histochimiques de cette tumeur rare.

## Patient et observation

**Figure 1:**
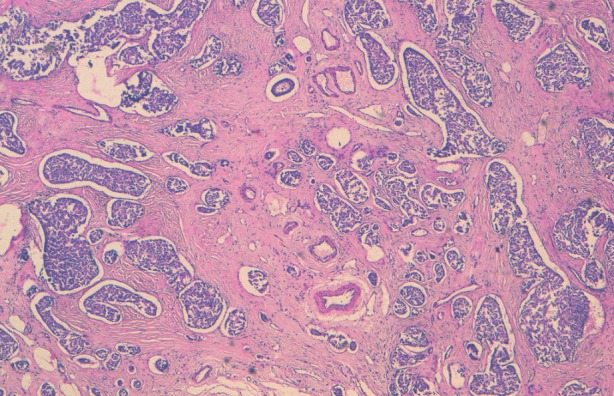
HESx10: Prolifération tumorale disposée en nids au sein d'un stroma fibreux

**Figure 2:**
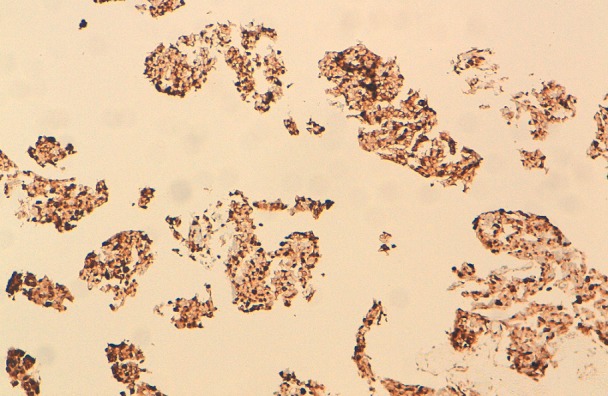
Les cellules tumorales expriment les marqueurs neuroendocrines (chromogranine)

**Figure 3:**
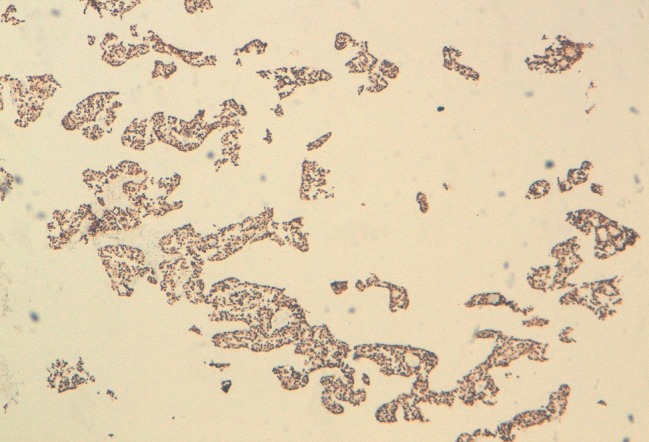
Les cellules tumorales expriment les récepteurs hormonaux

Notre cas concerne une patiente âgée de 50 ans, qui présente depuis un an un nodule du sein droit, d’évolution rapide, dure, sans adénopathie associée. Une biopsie de la tumeur a été réalisée. A l'examen histologique, il s'agissait d'une prolifération tumorale faite de nids de petites cellules rondes au noyau hyperchromatique et au cytoplasme mal limité amphophile (Figure 1). Focalement on notait la présence de vascularisation de type neuroendocrine. Il y'avait pas de foyers de carcinome canalaire in situ ni d'emboles vasculaire. L’étude immunohistochimique a confirmé la nature neuro-endocrine en montrant une expression de la chromogranine (Figure 2) et de la synaptophysine (Figure 3). par les cellules tumorales. Les récepteurs hormonaux étaient fortement positifs, la recherche de la surexpression d'HER2 était négative avec un index de prolifération KI67 à 5%.

## Discussion

Les carcinomes neuroendocrines sont des tumeurs rares. Leur incidence est estimée à 0,7 cas pour 100 000 habitants. Ils siégeant préférentiellement au niveau du tube digestif. La localisation mammaire est très rare [1, 2] représentant moins de 0.1% de tous les cancers mammaires et moins de 1% des tumeurs neuro-endocrines. [1] Ils touchent habituellement la femme âgée, entre la 6ème et la 7ème décade. L'homme peut également être touché [3–5]. L'existence de cellules neuroendocrines dans le parenchyme mammaire a été décrite en 1947 par Volger, et ce n'est qu'en 1977 que Cubilla et Woodruff ont décrit le premier cas de carcinome neuroendocrine primitif du sein [6]. D'autres rapports ont ensuite définis les caractéristiques macroscopiques, histologiques, histochimiques, immunohistochimiques ultrastructuraux, et moléculaires de cette tumeur. La présentation clinique de cette tumeur est similaire aux autres cancers du sein, sans signe spécifique en leur faveur. Elle se présente souvent sous forme d'un nodule palpable, bien circonscrit à la mammographie et à l’échographie [7]. La dernière édition de la classification OMS du cancer du sein publiée en 2003 reconnait le carcinome neuroendocrine comme une entité histologique à part entière, présentant les mêmes caractéristiques morphologiques que les tumeurs neuroendocrines du tractus gastro-intestinal, du pancréas et du poumon, avec expression d'un marqueur neuroendocrine par plus de 50% des cellules tumorales. La classification OMS exclue ainsi de cette définition les carcinomes mammaires (sans autre spécificité) avec différenciation neuroendocrine focale révélée par l'expression d'un marqueur neuroendocrine par des cellules éparses [8, 9] Cette différenciation neuroendocrine est rapportée dans environ 2-5% des cancers du sein [10]. Sur le plan histologique, la plupart des carcinomes neuroendocrines du sein se présentent sous forme de structures alvéolaires ou d'amas cellulaires solides d'aspect palissadique en périphérie. Selon le type cellulaire, le grade, le degré de différenciation et la présence d'une production de mucine, plusieurs sous-types sont définis dans la classification OMS: le carcinome neuroendocrine solide, le carcinome à petites cellules, dont les caractéristiques histologiques et immunohistochimiques sont identiques à celui du poumon et le carcinome neuroendocrine à grandes cellules [5, 11]. Le diagnostic de carcinome neuroendocrine repose sur l'expression d'un marqueur neuroendocrine par au moins 50% des cellules tumorales. La chromogranine et la synaptophysine sont les marqueurs neuroendocrines les plus sensibles et les plus spécifiques [11, 12]. D'autres marqueurs moins spécifiques peuvent également être exprimés (NSE, NCAM, neurofilament, bombésine). Les cytokératines de haut poids moléculaire sont négatives. L'expression de l'Her 2 est généralement absente tandis que les récepteurs oestrogéniques et progestéroniques sont fortement exprimés comme le cas de notre patiente [13, 14]. La coloration par le Grimelius est spécifique et montre une argyrophilie. L’étude ultrastructurale montre des granulations cytoplasmiques denses caractéristiques [10]. Des critères histologiques stricts ont été définis pour le diagnostic de carcinome neuroendocrine primaire du sein: la présence d'une composante in situ et/ou l'absence de localisation extra-mammaire [1]. Dans notre cas, il s'agit d'un carcinome neuroendocrine à petites cellules qui exprime, outre les marqueurs neuro-endocrines, et les récepteurs hormonaux. Le diagnostic différentiel peut se poser avec une métastase mammaire d'une tumeur carcinoïde d'autre origine. Le carcinome à petites cellules peut également être confondu avec un carcinome lobulaire; le marquage par l'E-cadhérine permet la distinction entre les deux tumeurs [16]. Le traitement des tumeurs endocrines du sein est surtout chirurgical. Les indications de la chimiothérapie et de la radiothérapie sont les mêmes que pour les autres cancers du sein. Les indications de l'hormonothérapie et de l'immunothérapie ne sont pas codifiées du fait que leurs effets restent incertains [16]. Le pronostic paraît aussi difficile à déterminer du fait du caractère exceptionnel de cette tumeur. Son comportement est inconnu. Toutefois, les carcinomes neuroendocrines non à petits cellules du sein traités semblent avoir un pronostic relativement favorable [17].

## Conclusion

Les tumeurs endocrines du sein sont des tumeurs rares, elles peuvent être primitives ou secondaires. Le diagnostic de certitude repose sur l’étude histologique, et plus particulièrement l’étude immunohistochimique. Les études concernant cette entité sont rares et regroupent un effectif réduit de cas. L’étude de séries plus larges permettra de mieux connaître leur histogenèse ainsi que leur profil évolutif.
